# *In vivo* acute toxicity and anti-gastric evaluation of a novel dichloro Schiff base: Bax and HSP70 alteration

**DOI:** 10.1093/abbs/gmz140

**Published:** 2019-12-30

**Authors:** Kamelia Saremi, Sima Kianpour Rad, Maryam Khalilzadeh, Jamal Hussaini, Nazia Abdul Majid

**Affiliations:** 1 Institute of Biological Science, Faculty of Science, University of Malaya, 50603 Kuala Lumpur, Malaysia; 2 Department of Molecular Medicine, Faculty of Medicine, University of Malaya, 50603 Kuala Lumpur, Malaysia; 3 Department of Plant Pathology, Institute of Food and Agricultural Sciences, University of Florida, Gainesville, FL 32611, USA; 4 Department of Medical Microbiology, Faculty of Medicine, Universiti Teknologi MARA 47000 Sungai Buloh, Selangor, Malaysia

**Keywords:** anti-gastric ulcer, Schiff base compound, CAT, SOD, HSP70, Bax

## Abstract

Chlorine is shown to possess anti-gastric ulcer activity, since it can inactivate *Helicobacter pylori*, which is regarded as one of the most common risk factors for causing gastric problems. In the current study, the gastroprotective property of a novel dichloro-substituted Schiff base complex, 2, 2′- [−1, 2-cyclohexanediylbis(nitriloethylidyne)] bis(4-chlorophenol) (CNCP), against alcohol-induced gastric lesion in SD rats was assessed. SD rats were divided into four groups, i.e. normal, ulcer control, testing, and reference groups. Ulcer area, gastric wall mucus, and also gastric acidity of the animal stomachs were measured. In addition, antioxidant activity of CNCP was evaluated and its safe dose was identified. Immunohistochemistry staining was also carried to evaluate two important proteins, i.e. Bcl2-associated X protein (Bax) and heat shock protein 70 (HSP70). Moreover, the activities of super oxide dismutase and catalase, as well as the levels of prostaglandin E2 (PGE2) and malondialdehyde (MDA) were also measured. Antioxidant activity of CNCP was approved via the aforementioned experiments. Histological evaluations showed that the compound possesses stomach epithelial defense activity. Additionally, periodic acid-Schiff staining exhibited over-expression of HSP70 and down-expression of Bax protein in the CNCP-treated rats. Moreover, CNCP caused deceased MDA level and elevated PGE2 level, and at the same time increased the activities of the two enzymes.

## Introduction

Gastric ulcer can cause sore in the gastrointestinal tract through developing vast injuries from the inside lining of ‘stomach’ to the upper portion of small bowel [1]. Several aggressive factors are able to provoke such disorder, including alcohol, aspirin, non-steroidal anti-inflammatory drugs (NSAIDs), and long run untreated *Helicobacter pylori* infection. Chronic gastritis is asymptomatic [2] and can be observed in NSAID-provoked ulcer, while upper gastrointestinal or peptic ulcer might be railed by *H. pylori* or aspirin consumption for a long time. NSAIDs or smoking can cause acid reflux, and therefore esophageal ulcer can be developed accordingly. Bleeding is one of the most common and severe complications of peptic ulcers [3], while perforation of gastric and duodenal walls are considered as the lowest incidences among all type of ulcers [4]. *H. pylori* and NSAIDs, or internal aggressive factors may lead to higher incidence of peptic ulcer via depleting the activity of defensive factors, including mucin, bicarbonate mucus barrier, and prostaglandins [5–7]. Anti-gastric medications are known to act through different molecular mechanisms and pathways. It was reported that heat shock proteins (HSPs), especially HSP70 could directly inhibit irritant-induced gastric ulcer formation [8]. It is known that gastric ulcer healing is a mechanism, which encompasses cell proliferation and migration at the gastric ulcer margin and angiogenesis in the granulation tissues [8–12]. It has been shown that HSP70, which is believed to be involved in primary folding and triage decisions, links molecular chaperones to autophagy and apoptosis. HSP70 can also accelerate gastric ulcer healing and is overexpressed in the damaged cells [10,13].

Conversely, apoptotic factors, such as Bcl2-associated X protein (Bax) proteins play crucial roles in the wound of acid-induced gastric ulcer due to its critical interplay in promoting cell survival, growth, proliferation, migration, and angiogenesis [12]. Previous studies have demonstrated that HSP70 is overexpressed along with downregulation of Bax, suggesting the inhibition of gastric ulcer production and promotion of wound and gastric ulcer healing [12,14,15].

Some chemicals and natural compounds possessing antioxidant property have anti-gastric ulcer activity, which mostly involves the inhibition of invasive acid secretion. On the other hand, some drugs, called cytoprotective alternative medicines, are able to defense stomach wall from detrimental factors without reducing stomach acid secretion [16].

Application of synthetic compounds with authenticated effectiveness against gastric ulcer is a promising approach to cure such complications. In this respect, Schiff bases, a substantial category of organic compounds, have broad applications in chemistry and medicine. Some prompt properties of such heterocyclic compounds are biological ‘properties*,*’ such as anticancer, antimicrobial, anti-inflammatory, and antioxidant activities [17–22]. Chelation of Schiff bases with different chemical substances, such as metal and halogens, showed higher biological activities compared with those without any substitutes [23]; therefore, substituted Schiff bases have better application as drugs and treatments. For instance, chlorine-substituted complex of Schiff bases showed prominent effect against gastric ulcer [24,25]. As a matter of fact, chlorine basically acts as a potent anti-inflammatory element and has significant capability to inhibit gastric ulcer by diminishing zinc-dependent endopeptidases expression and *H. pylori* growth in the gastric mucus. In our previous report, we showed that the same Schiff base-derived dibromo substituted compound, i.e. 2,​2′-​[​-​1,​2-​cyclohexanediylbis(n​itriloethylidyne)​]​bis(4-​bromophenol) (CNBP), possessed excellent anti-gastric ulcer activity [26].

Since chlorine has different anti-gastric and anti-inflammatory activities compared with bromine, in the present study, we decided to evaluate the anti-gastric ulcer activity of 2, 2′- [−1, 2-cyclohexanediylbis(nitriloethylidyne)] bis(4-chlorophenol) (CNCP). We synthesized a novel dichloro-substituted complex of Schiff base and evaluated its antioxidant activity. The gastro protective activity of the compound was assessed in rodent model by analyzing the effect of CNCP on the activities of antioxidant enzymes, the level reactive oxygen system (ROS), and the protein levels of Bax and HSP70.

## Materials and Methods

### Drugs

The necessary chemicals for synthesizing CNCP were purchased from Merck (Darmstadt, Germany) and Sigma-Aldrich (St. Louis, USA). SD rats were obtained from the Animal Experimental Unit, Faculty of Medicine, University of Malaya. The 3-(4,5-dimethylthiazol-2-yl)-2,5-diphenyltetrazolium bromide (MTT) reagent was purchased from USB Affymetrix (Cleveland, USA). Fibroblast cell line (BJ-5ta; ATCC^®^ CRL-4001™) was obtained from American Type Culture Collection (ATCC; Manassas, USA).

### Synthesis and characterization of CNCP

**Figure 1:**
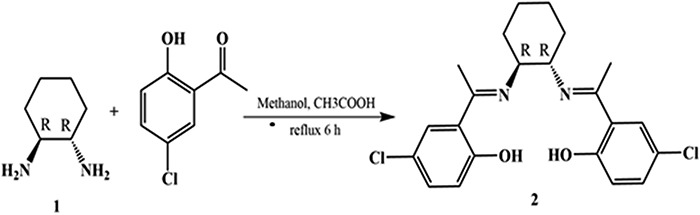
Synthesis of the Schiff base derivative CNCP

CNCP was synthesized according to the following protocol suggested by Yaul *et al.* [27]. A solution of trans-1,2-diaminocyclohexane (3.0 g, 26.27 mmol) in methanol (80 mL) was reacted with 5-chloro-2-hydroxyacetophenone (8.96 g, 52.54 mmol) under reflux condition for 6 h. After cooling to ambient temperature, yellowish solid crystals were collected by filtration, washed with methanol and dried over phosphorus pentoxide and recrystallized with ethanol to yield CNCP (8.82 g, 80%; **Fig. 1**). The compound was characterized with following data: m.p. 228–230°C. IR [KBr]: 3500 cm^−1^ (OH), 3010 cm^−1^ (CH_aromatic_), 2937, 2861 cm^−1^ (CH_aliphatic_), 1609 cm^−1^ (C=N), 1565 cm^−1^(C=C), 1256 cm^−1^ (C-N). ^1^H NMR (400 MHz, CDCl_3_): *δ* 7.34 (d, 2H, ^3^*J* = 2.6 Hz, 2× Ar-H), 7.16 (dd, 2H, ^3^*J* = 8.8 Hz, 2× Ar-H), 6.79 (d, 2H, ^3^*J* = 8.8 Hz, 2× Ar-H), 3.85 (dt~m_c_, 2H, 2× CH-N), 2.25 (s, 6H, 2× CH_3_), 1.9 (t, 4H, ^3^*J* = 9.5 Hz, 2× CH_2_-CH), 1.67 (p~m_c_, 2H, CH_2_), 1.48 (p~m_c_, 2H, CH_2_).^13^C NMR (100 MHz, CDCl_3_): *δ* 170.17 2× (C=N), 162.28 2× (Ar-OH), 132.40, 127.90 2× (CH_Ar_) 121.84 2× (Ar-Cl), 120.09 2× (CH_Ar_), 119.9 2× (C_Ar_-CN), 63.27 2× (CH-N), 32.32, 24.19 2× (CH_2_CH_2_), 14.55 2× (CH_3_).

### MTT assay

MTT assay was carried out on BJ-5ta cells (fibroblast cells) to determine the non-toxic dose of CNCP. Briefly, cells were cultured in DMEM supplemented with sodium pyruvate (110 mg), glucose (4500 mg), L-glutamine, 10% FBS, and 1% of a solution including two different antibiotics (penicillin and streptomycin). Culturing progress of the cells was conducted using the protocol described by Yaul *et al*. [27]. Percentage of cell growth was calculated using the following formula:

Cell viability (%) = (Absorbance of compound/Absorbance of control) × 100%.

### Ferric reducing antioxidant power

In ferric reducing antioxidant power (FRAP) assay, reduction of ferric tripyridyl triazine (Fe III TPTZ) into its ferrous form (blue color) was measured by the changes of absorbance at 593 nm. About 2.5 ml of 300 mM acetate buffer consisted of 100 ml H_2_O_2_, 1.6 ml glacial acetic acid and 0.31 g sodium acetate was added to 1 ml of 1 mg/ml of stock solution, which then followed by addition of 2.5 ml TPTZ solution, which made by adding 0.0625 g to 20 ml H_2_O_2_ to the mixture. At 4 min intervals, the absorbance was measured at 593 nm. Gallic acid and ascorbic acid were selected as standard and positive controls, respectively.

### DPPH radical scavenging assay

The 1,1-diphenyl-2-picrylhydrazyl (DPPH) radical scavenging activity was carried out using the method described by Ghasemzadeh *et al.* [28]. A total of 0.6 ml DPPH radical solution (0.004 g of DPPH reagent in 100 ml methanol) was added to 100 μl of diluted stock solution (DPPH reagent), followed by incubation in the dark for 20 min. The absorbance was measured at 517 nm. Ascorbic acid was used as the reference.

### Animal grouping and ethical statement

Forty-eight adult male SD rats (200–250 g) were kept in separate plastic cages at 23 ± 2°C under half-light/half dark cycle and allowed to access to water *ad libitum* and standard chow pellets. The rats were ‘acclimated’ in standard ‘laboratory animal room condition’ for 24 h before the experiments with free access to food only, but no water. All the experimental procedures were approved by the Ethic Committee of the Research Center, University of Malaya; Council on Animal Care Guideline (Ethic No. 2015-09-11/BMS/R/MAA).

### Acute toxicity

Eighteen female SD rats were randomly assigned into three groups with six rats in each group: Control (vehicle) group which was orally fed with 10% Tween 20, LD group which was fed with low dose of CNCP (100 mg/kg), and HD group which was fed with high dose of CNCP (200 mg/kg). Only food was available for animals in both cycles overnight and after starvation. The animals were fed with the compound orally by gavage and then the toxicity evaluation of CNCP was performed at 30 min, 2, 4, 24, and 48 h. Animal behaviors and mortality rate were checked after 2 weeks of feeding [29,30]. The animals were euthanized by ketamine and xylazine at a dosage of 10 mg/kg, and the blood samples collected from the cardiac punctures were subject to serum biochemical profiling. Finally, histopathology study was carried out on the liver and kidney of the animals [31].

### Ethanol-induced gastric ulcer

The SD rats were classified into five groups, each contained six animals. Both normal and ulcer groups were administered with 10% Tween 20 (5 ml/kg) after 24 h of starvation. Control group was fed with 20 mg/kg omeprazole in volume of 5 ml/kg, and the testing groups received two different doses of CNCP: 10 mg/kg for the LD group and 20 mg/kg for the HD group, respectively. The animal groups, except the normal group, were orally fed with absolute ethanol by gavage to induce stomach injuries [32–34]. After 1 h, animals were euthanized and their stomachs were removed for subsequent experiments [35].

### Evaluation of gastric fluid acidity

Each stomach was carefully cut from the bigger curvature in order to collect the juice. The supernatant was obtained by centrifugation at 1008 *g* for 10 min and tested for the pH [36].

### Determination of gastric wall mucus

Gastric wall mucus (GWM) determination was done based on the method developed by Corne *et al*. [37]. The glandular portions of the stomachs were firstly weighed and immediately mixed with 10 ml of 1% (w/v) Alcian blue staining solution (0.16 M of sucrose solution and 0.5 ml, sodium acetate, pH 5). After 2 h, the excessive dye was washed away by two times rinse with 10 ml of 0.25 M of sucrose. Then, the Alcian blue dye attaching to the stomach wall mucus was completely removed by incubation with 10 ml of 0.5 M of magnesium chloride for 30 min. About 4 μl of the product was mixed with 4 ml of ethyl ether and shaken for 120 s, followed by centrifugation at 1008 *g* for 10 min. The absorbance of the supernatant was measured at 598 nm and the amount Alcian blue extracted from 1 g of glandular stomach tissue was calculated using the formula as described previously [29].

### Measurement of ulcer area

The length and width (mm) of each hemorrhagic lesion of the animals were measured using a planimeter (10 × 10 mm  =  ulcer area [UA]) under a dissecting microscope (magnification =1.8×). The sum of the area of the lesions for each stomach was used to calculate the UA [38] and UI was calculated using the following formula:

$$ \mathrm{UI}\ \left(\%\right)=\left[\left(\mathrm{UA}\ \mathrm{of}\ \mathrm{C}\hbox{--} \mathrm{UA}\ \mathrm{of}\ \mathrm{T}\right)/\mathrm{UA}\ \mathrm{of}\ \mathrm{C}\right]\times 100\% $$

where UI is the ulcer inhibition, T is the treatment, and C is the negative control.

### Preparation of gastric homogenates

The tissues were homogenized according to a method described by Sidahmad *et al*. [39]. Homogenization of tiny segments of glandular portion of each stomach was done in 50 mM PBS (pH = 7.2) at 4°C with a teflon homogenizer (Polytron, Heidolph RZR 1, Schwabach, Germany), followed by centrifugation at 2580 *g* for 15 min. The supernatant was used for measuring the activities of catalase (CAT) and super oxide dismutase (SOD), the levels of malondialdehyde (MDA) and prostaglandin E2 (PGE2), as well as the expressions of HSP70 and Bax proteins.

### Measurement of stomach’s protein concentration

Protein concentration of the homogenate stomach tissues (1 mg/ml) was measured according to the Biuret reaction [40].

### Measurement of SOD and CAT activity

Enzymes activity measurement was performed using the Cayman Chemical SOD and CAT Assay kits (Cayman Chemical Co., Ann Arbor, USA) according to the manufacturer’s instruction.

### Gastric level of MDA and PGE2

Gastric levels of PGE2 were detected using the Cayman PGE2 monoclonal enzyme immunoassay kit. In addition, to estimate the grade of lipid peroxidation in the gastric mucous membrane, the level of MDA was measured using Cayman TBARS kit.

### Hematoxylin and eosin staining

Phosphate buffered formalin (10%) was used to fix specimens of the stomach’s wall at ambient temperature. Then samples were subject to tissue-processing (dehydration, clearance, and infiltration with paraffin) on a tissue-processing machine (Leica, Solms, Germany), followed by paraffin-embedding. The stomach tissues were sectioned at a thickness of 5 μm, and stained with hematoxylin and eosin (H&E) for further histological study [41].

### Gastric mucosal glycoprotein evaluation

Segments of each stomach’s wall was stained with periodic acid-Schiff (PAS) in order to obtain clear observation of gastric epithelial mucus secretion and better evaluation of any changes in either acidic or basic glycoproteins [42].

### Immunohistochemically stain

Immunostaining of Bax and HSP70 was done according to the manufacturer’s instruction of Dako kits (Dako Cyomation, Carpineteria, USA).

### Statistical analysis

All data are shown as the mean ± SEM. Differences among the experimental groups were determined by one-way ANOVA followed by Tukey’s *post-hoc* test for multiple comparisons using SPSS version 24. Values of *P* < 0.05 were considered as significant.

## Results

### Cytotoxicity of CNCP

**Figure 2:**
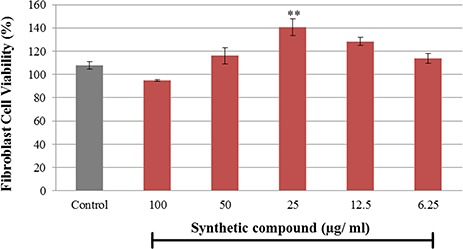
**The effect of CNCP compound on fibroblast cell viability** The data are presented as the mean ± SEM. ^**^P < 0.01 with the untreated cells (control).

The influence of CNCP on human fibroblast cell proliferation was determined by MTT assay (**Fig. 2**). It was found that the cell proliferation was promoted after treatment with CNCP, suggesting that the compound affected positively on the proliferation and viability of fibroblast cells. The threshold concentration for significant increase of cell proliferation was noticed at 12.5–25 μg/ml.

### Antioxidant activity of CNCP evaluated by both FRAP and DPPH

**Figure 3:**
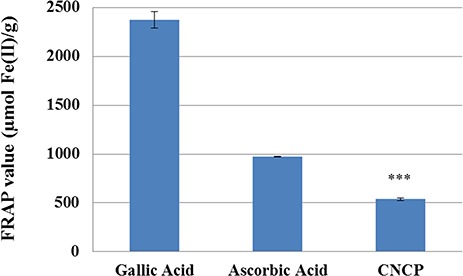
**Comparison of the FRAP value of CNCP with the synthetic reference standard (ascorbic acid and gallic acid)** Data are presented as the mean ± SEM (*n* = 3). ^***^P < 0.001.

Capability of CNCP in reducing ferric tripyridyl into ferrous form was noticeable (**Fig. 3**). FRAP value of the compound was 536.9 ± 11.9 μmol Fe (II)/g, which was significantly lower than those of ascorbic and gallic acids with values of 973.7 ± 3.5 and 2373.8 ± 84.7 μmol, respectively.

**Figure 4:**
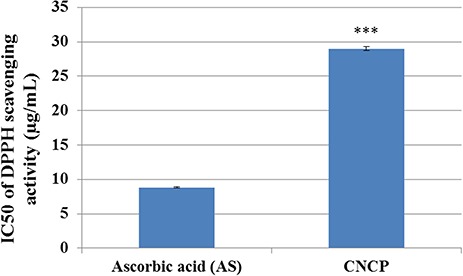
**IC_50_ of the DPPH-scavenging activity of CNCP compound and ascorbic acid** All the values were presented as the mean ± SEM (*n* = 3). ^***^P < 0.001. Ascorbic acid was used as a reference.

Furthermore, the DPPH-scavenging activity of CNCP is shown in **Fig. 4**. The results indicated that the activity of the compound was higher than that of ascorbic acid, as verified by the IC_50_ values (29 μg/ml for CNCP and 8 μg/ml for ascorbic acid).

### Acute toxicity of CNCP

**Figure 5:**
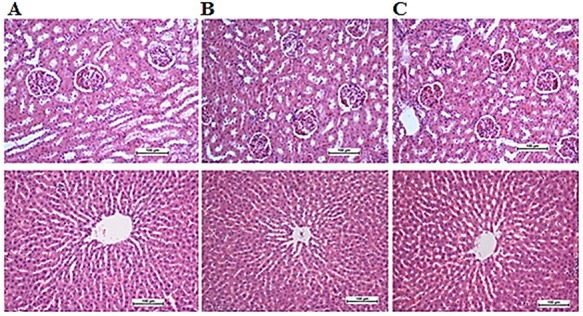
Histological sections of kidney and liver tissues of SD rats (*n =* 6) (A) Vehicle control (10% Tween 20), (B) (100 mg/ml), and (C) CNCP (200 mg/ml) in SD rats. No structural difference was detected among the CNCP-treated and control groups. Upper row: kidney, lower row: liver.

Effects of CNCP on renal and liver functions and lipids profile of the rats are listed in **Tables 1–3**. Any abnormalities in physiological feature or behavioral changes were detected in the animals. The changes in body weight during the time of monitoring at the doses used in 2 weeks were noticed, but no mortality or signs of toxicity were detected. Histological examination of the liver, kidney, and the serum biochemical analysis in treated groups showed that there was no significant difference compared with those of the control group (**Fig. 5**).

**Table 1:** Effect of CNCP on renal function of SD rats (*n* = 6)

Renalfunction test	Animal group		
	Control (10% Tween 20)	CNCP (100 mg/kg)	CNCP (200 mg/kg)
Sodium (mM)	144.050 ± 0.33	143.63 ± 0.25	141.68 ± 0.56
Pottasium (mM)	4.50 ± 0.15	4.56 ± 0.36	4.59 ± 0.18
Chloride (mM)	103.85 ± 0.28	104.66 ± 1.14	102.01 ± 1.21
CO_2_ (mM)	25.02 ± 0.91	27.35 ± 1.18	28.05 ± 0.73
Anion (mM)	18.00 ± 0.93	15.97 ± 1.50	16.84 ± 1.84
Urea (mM)	7.01 ± 0.75	6.43 ± 0.84	7.62 ± 1.07
Creatinine (μM)	36.37 ± 1.92	37.33 ± 1.37	38.05 ± 1.72

All values are displayed as the mean ± standard error mean. No significant differences were detected between the groups. The significant value at *P* < 0.05 was considered.

**Table 2:** Effect of CNCP on liver function in SD rats (*n* = 6)

Liver function test	Animal group		
	Control (10% Tween 20)	CNCP (100 mg/kg)	CNCP (200 mg/kg)
Total protein (g/l)	61.17 ± 0.98	59.05 ± 0.47	60.01 ± 2.05
Albumin (g/l)	40.09 ± 0.49	32.37 ± 1.65	38.03 ± 0.95
Globulin (g/l)	21.67 ± 0.56	19.06 ± 1.92	22.11 ± 1.26
Total bilirubin (μM)	2.00 ± 0.15	2.01 ± 0.17	2.02 ± 0.15
Conjugatedbilirubin (μM)	1.05 ± 0.10	1.04 ± 0.08	1.01 ± 0.12
Alkalinephosphatase (IU/l)	150.65 ± 0.71	147.97 ± 9.78	143.81 ± 2.73
Alaninetransaminase (IU/l)	49.35 ± 5.71	48.05 ± 4.62	49.33 ± 3.27
Aspartatetransaminase (IU/l)	169.63 ± 2.94	167.39 ± 3.61	170.13 ± 4.03
G-Glutaml. transferase (IU/l)	1.93 ± 0.21	2.02 ± 0.02	2.00 ± 0.02

All values are displayed as the mean ± standard error mean. No significant differences were detected between the groups. The significant value at *P* < 0.05 was considered.

**Table 3:** Effect of CNCP on Lipid profile in SD rats (*n* = 6)

Lipid profile analysis	Animal group		
	Control (10% Tween 20)	CNCP (100 mg/kg)	CNCP (200 mg/kg)
Triglyceride (mM)	0.33 ± 0.03	0.31 ± 0.03	0.39 ± 0.05
Total cholesterol (mM)	1.43 ± 0.15	1.33 ± 0.13	1.30 ± 0.22
HDL cholesterol (mM)	1.45 ± 0.06	1.39 ± 0.05	1.38 ± 0.04
LDL cholesterol (mM)	0.81 ± 0.10	0.65 ± 0.10	0.73 ± 0.11

All values are displayed as the mean ± standard error mean. No significant differences were detected between the groups. The significant value at *P* < 0.05 was considered.

### Effect of CNCP on the pH of gastric secretion and GWM

**Figure 6:**
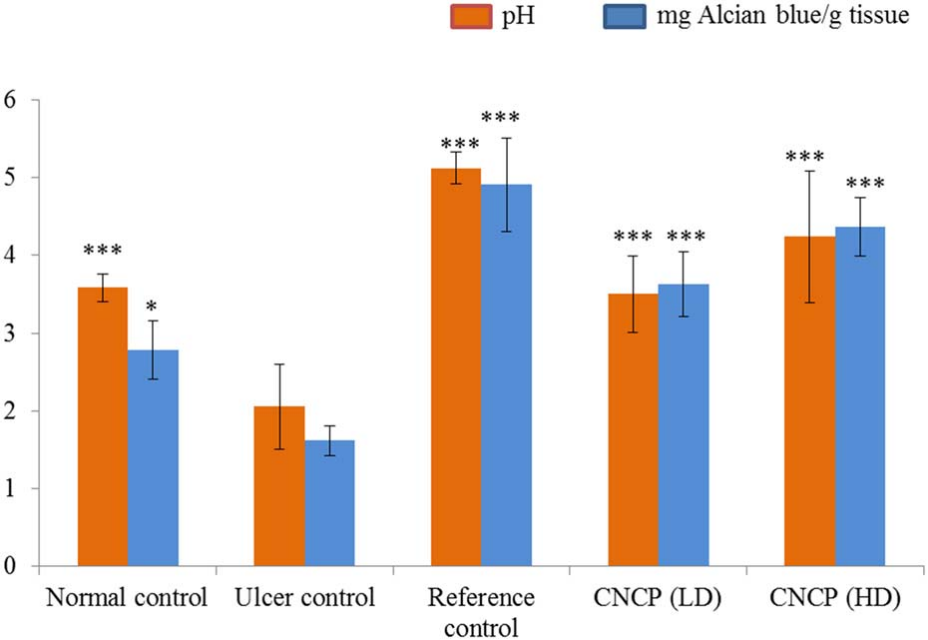
**Effect of CNCP compound on pH and GWM** Data are presented as the mean ± SEM (*n* = 6). ^*^P < 0.05 and ^***^P < 0.001. Reference group was given 20 mg/kg omeprazole, low dose (LD; 10 mg/kg) and high dose (HD; 20 mg/kg) of CNCP.

*In vivo* confocal microscopy was used to make sure that the rats were stable enough during the preparation for the subsequent experiments. In addition, temperature of the animal’s body was recorded as 36 ± 0.5°C and 35.5 ± 0.2°C, before and after 3–4 h of the confocal stage, respectively. In experimental animals pretreated with omeprazole and CNCP (10 and 20 mg/kg), the pH of the gastric contents was significantly increased compared with that of the ulcer control group (**Fig. 6**). Treatment with ethanol could significantly decrease the mucus content of the stomach wall in the ulcer group when compared with the normal animals. The collapsed gastric mucus content could be remarkably (^***^P < 0.001) restored in the animals treated with CNCP (**Fig. 6**).

### Effect of CNCP on stomach mucosa

**Figure 7:**
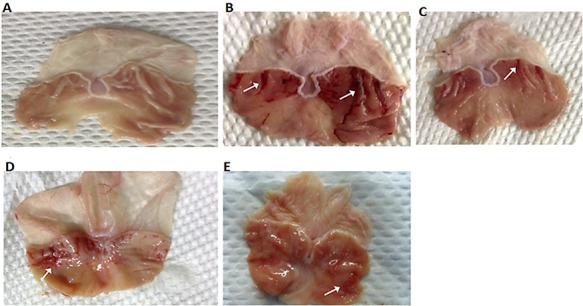
Effect of CNCP on gross images of absolute ethanol-induced gastric injury in rats (*n* = 6) (A) Normal control gastric epithelium (10% Tween 20). (B) Ulcerated stomach (absolute ethanol) exhibiting extraordinary acute hemorrhagic ulceration (white arrow). (C) Reference class (omeprazole, 20 mg/kg) exhibiting mild injury, (D, E) stomachs pre-fed with CNCP compound at low dose (LD, 10 mg/kg) and high dose (HD, 20 mg/kg), respectively showing noticeable reduction in the gastric lesions.

**Figure 8:**
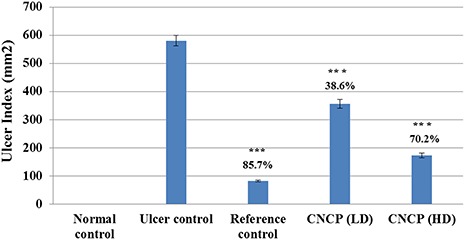
**UA calculation of CNCP compound for both doses** Values are presented as the mean ± SEM (*n* = 6). ^***^P < 0.001. Animals pre-fed with CNCP compound significantly reduced UA compared to ulcer control rats. Percent (%) of ulcer inhibition of the reference and experimental groups are shown above the bars.

Total stomach signs of those rats administered with CNCP showed remarkable decrease in the red severe ulcerated bands, which coexisted with acute inflammation when it was compared with that of the ulcer control animals (**Fig. 7**). The effect of CNCP on the UA induced by ethanol is depicted in **Fig. 8**. In fact, this appearance was observed in the UA measured as the percentage of inhibition. However, CNCP-pre-fed with both LD and HD of CNCP could drastically reduce UA and the percentage of inhibition compared with the ulcer group, and high dose of CNCP showed the highest reduction of UA (**Fig. 8**).

### Gastric antioxidant activity of CNCP

The effects of CNCP on endogenous antioxidant enzymes, SOD, and CAT are listed in **Table 4**. Because of the ethanol insults in ulcer control groups, significant reductions of SOD and CAT activities in comparison with control group were found. Stomach of the rats treated with high dose (20 mg/kg) of CNCP could significantly increase those activities when compared with the ulcer control group (**Table 4**). CNCP (both LD and HD), significantly decreased SOD and CAT activities when compared with the reference control group.

**Table 4:** **Effect of CNCP on endogenous antioxidant enzymes activity, levels of MDA and PGE**_**2**_, and protein concentration

Animal group	SOD (U/mg protein)	CAT (nM/min/ml protein)	MDA (μM/g protein)	PGE_2_ (ng/mg protein)	Protein concentration (mg/ml)
10% Tween 20 (Normal control)	17.62 ± 0.75^***^	84.91 ± 2.12^***^	60.31 ± 2.70^***^	3.1 ± 0.17^***^	9.03 ± 0.15^***^
Absolute EtOH (Ulcer control)	4.06 ± 0.51	18.57 ± 0.03	142.65 ± 0.11	1.09 ± 0.01	5.15 ± 0.47
Omeprazole (20 mg/kg)	20.11 ± 0.51^***^	100.55 ± 0.36^***^	80.95 ± 2.52^***^	2.97 ± 0.05^***^	7.38 ± 0.87^**^
CNCP (10 mg/kg)	13.54 ± 0.65^***^	54.95 ± 4.57^**^	128.81 ± 8.06	2.10 ± 0.22^***^	6.49 ± 0.01
CNCP (20 mg/kg)	19.21 ± 0.85^***^	70.73 ± 4.55^***^	110.48 ± 5.15^*^	2.50 ± 0.13^***^	7.32 ± 0.06^*^

All values are expressed as the mean ± SEM. ^*^P < 0.05, ^**^P < 0.01, and ^***^P < 0.001 compared with the ulcer group.

### Effect of CNCP on MDA, PGE2, and protein levels in gastric tissue homogenates

The effect of CNCP on MDA and PGE2 levels are listed in **Table 4**. Ethanol could elevate the level of lipid peroxidation in the ulcer group when compared with the normal group, as indicated by the high level of gastric MDA. Like omeprazole, the two doses of CNCP could strongly decrease MDA level compared with the ulcer control group. The PGE2 level in the alcohol-treated animals was notably lower than that in the normal control group. CNCP could significantly elevate PGE2 level when compared with the ulcer control group.

Meanwhile, CNCP (HD) could significantly elevate the total protein level in gastric homogenates compared with the ulcer control group (**Table 4**).

### CNCP possesses significant gastroprotective activity

**Figure 9:**
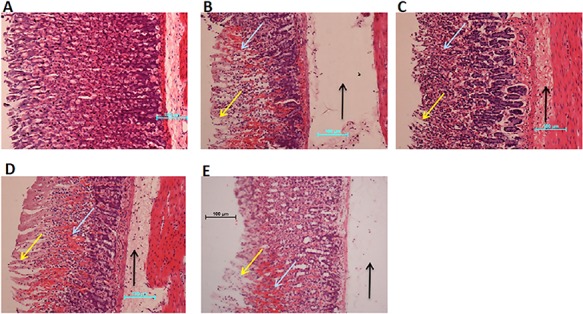
Effect of CNCP compound on histology of gastric epithelium in ethanol-induced gastric mucosal damage in rats (*n* = 6) (A) Normal control rats. (B) Ulcer control stomach showing severe mucosal injury (yellow arrow) along with deep necrosis (blue arrow), edema, and inflammation of sub-mucosal layer (black arrow). (C) Reference control stomach (omeprazole, 20 mg/kg) showing mild mucosal injury. (D, E) Experimental animals stomachs pre-fed with CNCP (LD, 10 g/kg and HD, 20 mg/kg). High dose of CNCP had better gastroprotective effect than low dose of CNCP.

Images of tissue sections stained with H&E are shown in **Fig. 9**. Ethanol in gastric lesions could cause significant damage of the gastric mucosal epithelium. In addition, the deep lesions (blue arrow) showed obvious necrosis of the mucosa together with extensive edema (black arrow) and remarkable inflammation in the ulcer animals. CNCP (HD) could protect the gastric mucosa of animals with sign of reduction of the ulcer lesions (**Fig. 9**).

**Figure 10:**
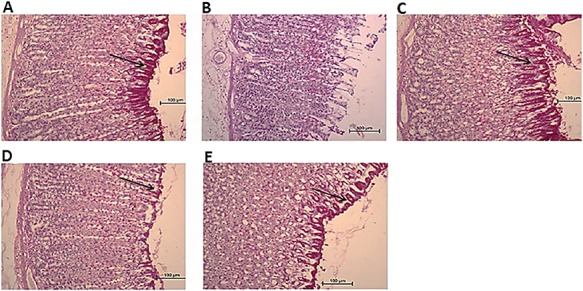
Effect of CNCP on gastric glycoprotein secretion in ethanol-induced stomach damage in rats (*n* = 6) (A) Normal control class exhibiting normal magenta color (black arrow) of gastric mucus glands. (B) Absence of PAS staining from mucosa of ulcer control class exhibiting severe mucosal injuries. (C) Reference group exhibiting intense PAS stain. (D, E) Experimental groups fed with low dose (10 mg/kg) and high dose (20 mg/kg) of CNCP compound respectively exhibiting intense uptake of PAS stain. High dose of CNCP compound (E) showed more intense PAS staining than low dose of CNCP compound (D).

As depicted in **Fig. 10**, PAS staining showed reduction of gastric mucosal secretion in the ulcer group. The positive PAS staining of the mucosal lining of the stomach in the CNCP HD-treated group showed higher level of mucosal glycoproteins than those of the ulcer group. These findings suggested that CNCP possesses significant gastroprotective activity.

### Effect of CNCP on the protein expressions of HSP70 and Bax

**Figure 11:**
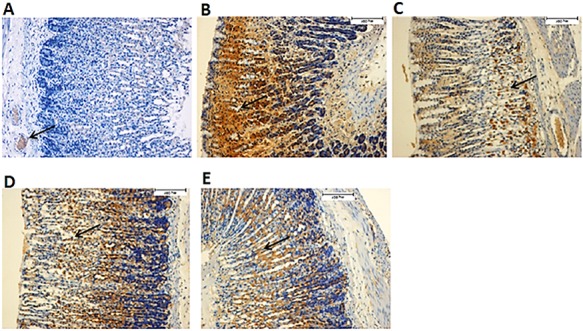
Effect of the CNCP on Bax protein expression in ethanol-induced gastric epithelial damage in rats (*n* = 6) (A) Normal control group. (B) Ulcer control group showing up-regulated Bax protein (black arrow). (C) Reference control group showing clear down-regulation of Bax protein. (D, E) Rats pre-treated with low dose (10 mg/kg) and high dose (20 mg/kg) of CNCP compound respectively showing down-regulation of Bax protein.

**Figure 12:**
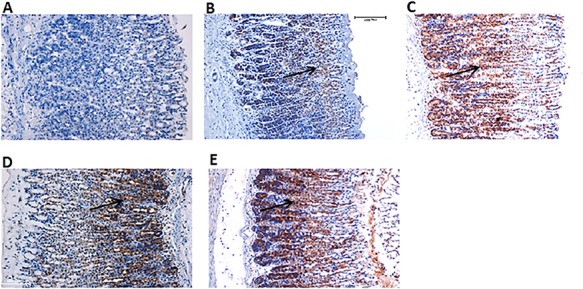
Effect of CNCP compound on HSP70 protein expression in gastric mucosa of ethanol-induced stomach ulcer in rats (*n* = 6) (A) Normal control group. (B) Ulcer control group expressed less HSP70 protein (black arrow). (C) Reference group demonstrated obvious up-regulation of HSP70 protein. (D, E) Experimental groups pre-fed with CNCP compound (10 and 20 mg/kg, respectively) exposing higher expression of HSP70 protein in high dose-CNCP fed group (E) better than low dose group (D).

Images of tissue sections subject to immunohistochemical staining for the HSP70 and Bax are shown in **Figs. 11**, **12**. In the CNCP HD-treated rats, over-expression of HSP70 was noticed compared with the ulcer control group. Staining for the Bax protein showed that ethanol could induce injury and apoptosis in the stomachs with overexpression of Bax, while pre-treatment with CNCP HD caused down-regulation of Bax expression.

## Discussion

*Gastric ulcer* is the deterioration of extensive necrotic lesion encompassing whole mucosal vastness and muscularis *mucosa* [43] and can be developed in any imbalanced conditions in the gastric system. Such undesirable conditions can happen when the mucosal defensive system is affected by some detrimental factors, such as, high acidity, infection, and other factors on the luminal ‘surface *of* stomach’ [1]. It causes puncture or/and acute hemorrhagic ulcer; however, there are some internal restrictive factors, such as PGE2, mucus secretion, and bicarbonate synthesis, which can inhibit ulcer progression. Despite the major role of defensive system against any ulcer in the body, some imbalanced conditions between self-protective agents and external or internal damaging factors, including extra stomach acid, *H. pylori* infection and its proteolytic enzymes, ethanol consumption, cigarettes and etc. lead to peptic ulcer formation [29]. It has also been found that generation of free radicals, which are initiated by such damaging factor plays key roles in the formation and progression of the disease [34].

Some previous studies showed direct correlation between suppression of extra acid production in the stomach and the effective treatments [26,43]. Due to the crucial role of extra acid neutralization in stomach by lowering the activation of acid producer pumps and the prominent role of oxidants in the production of extra acidity, antioxidants can play key roles in inhibiting gastric ulcer and the relative diseases [26,43].

In the present study, CNCP was found to increase the generation of epithelial cells, which could drastically increase protein concentration in the gastric secretions of the pre-treated animals.

Absence of any sign of toxicity and mortality during the experiments suggests that the compound at 100 and 200 mg/kg was safe to use. Furthermore, neither hepatic nor renal toxicity were detected in the rats treated with CNCP. Antioxidant activity of CNCP was detected by both DPPH and FRAP evaluations. It has been demonstrated that metabolism of ethanol in the body could generate ROSs, such as superoxide anions and/or hydroperoxy, leading to both acute and chronic gastrointestinal ulcer [44,45]. Several factors can contribute to the formation of ethanol-induced gastric wounds, such as gastric mucus reduction and over production of free radicals. Such factors, individually or maybe together, lead to lipid peroxidation, resulting in the damage of surface layer of mucosal epithelium [46]. CNCP showed significant effect against acute hemorrhagic lesions of gastric mucosa induced by ethanol. It was found that gastric anti-ulcer activity of CNCP could be due to its ability for maintaining stomach mucus discharge, leading to decrease of mucosa volume and increase of surface mucosal area.

CNCP was found to have significant anti-ulcer effect via increasing both pH and mucus and decreasing sub-mucosal edema and sub-mucosal inflammation. Such findings were in agreement with several other studies, which evaluated the gastroprotective and anti-ulcer activity of several novel synthetic compounds [33,47]. It was found that gastric mucus functions, such as inhibition of over production and secretion of acid and pepsin, were considered as notable defensive factors against any injuries of gastrointestinal tract [29]. In the present study, it was noticed that CNCP could considerably increase the stomach mucus secretion and decrease the acidity of stomach contents. Additionally, the compound could increase the glycoprotein of glandular portion of surface epithelium, which was confirmed by PAS staining. Similarly, the results of some previous studies demonstrated significant incline of gastric mucus secretion in rats pre-treated with different synthetic compounds [33,34,48,49]. The findings showed that gastroprotective effect could be resulted from the preservation of mucus secretion. Progression of gastric mucosal damage could be developed by stress, causing drastic increase in stomach’s acid secretion and leading to mucosal layer damage, so it could not be protected by suppressing mucus production [40, 41].

CNCP-pre-treated animals showed considerable decline in MDA level compared with ulcer control group animals, which can be caused by the capability of the compound to increase the activation of the antioxidant enzymes. Likewise, reduction of MDA level and induction of activities of SOD and CAT were suggested to be the most important factors associated with the gastro-protective effect of drugs [50]. It was also noticed in our study that those rats fed with CNCP showed significant increase in both SOD and CAT activities, which were coupled with the reduction of gastric MDA level. Our results were in agreement with those reports indicating that the activities of SOD and CAT were increased in the synthetic compound-treated rodents [34,51–53].

Some previous studies suggested that PGE2 can be inactivated when free radicals are increased, leading to mucosal damage. Significant role of PGE2 in regulating mucus secretion from the stomach walls has been proved and found to possess defensive activity in different gastric wound models [5,54]. Takeuchi *et al.* [55] found the mechanism of action of prostaglandins in motivating mucus production and bicarbonate discharging. This could sustain mucosal blood circulation, leading to the protection of epithelial cells from any damage induced by some inflammatory elements, such as cytotoxins. Hajrezaie *et al*. [56] found that the gastroprotective action of prostaglandins against stomach mucosal damage could be initiated by the preservation of gastric mucus productions. Based on the results of the current study, it is noticed that enhancement of the mucosal level of PGE2 in CNCP-treated rats can partly support the gastroprotective effect of the compound.

Ibrahim *et al*. [57] noticed that the HSP70 protein level was increased in response to either stress or high generation of ROS. In fact, the ethanol-induced ROS generally can damage proteins, causing partial unfolding and aggregation. CNCP could protect gastric tissues via up-regulation of HSP70, which inhibits tissues from aggregation. In addition, Singh *et al.* [58] suggested that HSP70, as an important apoptosis regulator protein, can regulate both adaptive and internal immune reactions. In fact, the HSPs family, particularly HSP70, can interplay with both up- and down-stream apoptotic-mediating elements against stress condition via cytoprotection. In peptic ulcer, for instance, it was found that HSP70 inducers could protect gastric mucosa against ethanol-induced injury through rehabilitation of HSP70 expression in gastric wall cells in animal model. Moreover, HSP70 protein possesses conservative effect on usual construction of proteins and also shows excellent ability to abolish toxic remains of cell lysis [59]. Similarly, up-regulation of HSP70 protein in the testing animals showed that CNCP possessed gastroprotective effect by enhancing HSP70 expression in gastric tissues of the treated rats.

The pro-apoptotic protein, Bax, as a member of Bcl-2 family, is associated with the regulation apoptosis through mitochondrial damages [60,61]. In fact, ethanol can trigger the induction of apoptosis in gastric epithelium by overexpression of pro-apoptotic proteins, such as Bax and/or down-expression of anti-apoptotic bodies, like Bcl-2 [62]. Bax protein was found to be down-regulated and HSP70 up-regulated in the gastric tissues of those animals administered with CNCP when compared with those in the ulcer control group. Our findings were in agreement with the results of some previous studies indicating that induction of HSP70 protein along with suppression of Bax protein in animals can cause protection of gastric mucosa against damages induced by ethanol [29,34,45,63].

In summary, CNCP is safe for use and it has significant gastro-protective potential via the protection of stomach epithelium against ethanol-induced damage. CNCP could drastically enhance the activities of SOD and CAT, while it could retard the level of MDA in stomach tissue homogenates and could remarkably elevate the level of PGE2. The significant increase of GWM in CNCP-treated rats suggested the protective capacity of the compound. Reduction of hemorrhagic mucosal lesions in stomach mucosal epithelium and decrease of edema and leukocytes penetration of sub-mucosal layers were also observed in CNCP-treated animals. CNCP could increase glycoprotein contents, and moreover it could cause up-regulation of HSP70 protein and down-regulation of Bax protein, indicating its effectiveness in regulating apoptosis. The present study provides convincing evidence that CNCP possesses gastroprotective activity in gastric ulcer models via the pathways, leading to the increase of the levels of mucus and PGE2, as well as the activities of SOD and CAT.

## References

[1] Padmavathi G, JN NSC, Shampalatha S. Antiulcer activity of *Trigonella foenum*-graecum leaves in cold restraint stress-induced ulcer model. Molecular & Clinical Pharmacology 2012, 3: 90–91.

[2] He C, Yang Z, Lu N Imbalance of gastrointestinal microbiota in the pathogenesis of helicobacter pylori-associated diseases. Helicobacter 2016, 21: 337–348. 26876927 10.1111/hel.12297

[3] Xi B, Jia J-J, Lin B-Y, Geng L, Zheng S-S Peptic ulcers accompanied with gastrointestinal bleeding, pylorus obstruction and cholangitis secondary to choledochoduodenal fistula: a case report. Oncol Lett 2016, 11: 481–483. 26870237 10.3892/ol.2015.3908 PMC4727103

[4] Pakodi F, Abdel-Salam OM, Debreceni A, Mózsik G Helicobacter pylori. One bacterium and a broad spectrum of human disease! An overview. Journal of Physiology-Paris 2000, 94: 139–152. 10.1016/s0928-4257(00)00160-1 10791696

[5] Golbabapour S, Hajrezaie M, Hassandarvish P, Abdul Majid N, Hadi AHA, Nordin N, Abdulla MA Acute toxicity and gastroprotective role of *M. pruriens* in ethanol-induced gastric mucosal injuries in rats. Biomed Res Int 2013, 2013: 974185. 23781513 10.1155/2013/974185 PMC3678452

[6] Indran M, Mahmood A, Kuppusamy U Protective effect of *Carica papaya* L leaf extract against alcohol induced acute gastric damage and blood oxidative stress in rats. West Indian Medical Journal 2008, 57: 323–326. 19566009

[7] Wasman S, Mahmood A, Chua LS, Alshawsh MA, Hamdan S. Antioxidant and gastroprotective activities of *Andrographis paniculata* (Hempedu Bumi) in Sprague Dawley rats. Indian Journal of Experimental Biology 2011, 49: 767–772. 22013743

[8] Suemasu S, Tanaka K-I, Namba T, Ishihara T, Katsu T, Fujimoto M, Adachi H, et al. A role for HSP70 in protecting against indomethacin-induced gastric lesions. J Biol Chem 2009, 284: 19705–19715. 19439408 10.1074/jbc.M109.006817 PMC2740595

[9] Ishihara T, Suemasu S, Asano T, Tanaka K-i, Mizushima T Stimulation of gastric ulcer healing by heat shock protein 70. Biochem Pharmacol 2011, 82: 728–736. 21736872 10.1016/j.bcp.2011.06.030

[10] Park JM, Kim JW, Hahm KB HSPa4, The “Evil Chaperone” of the HSP Family, Delays Gastric Ulcer Healing. Digestive Diseases and Sciences 2015, 60: 824–826. 25732714 10.1007/s10620-015-3597-9

[11] Sidahmed HM, Hashim NM, Amir J, Abdulla MA, Hadi AHA, Abdelwahab SI, Taha MME, et al. Pyranocycloartobiloxanthone a, a novel gastroprotective compound from *Artocarpus obtusus* Jarret, against ethanol-induced acute gastric ulcer in vivo. Phytomedicine 2013, 20: 834–843. 23570997 10.1016/j.phymed.2013.03.002

[12] Mohamed WA, Abd-Elhakim YM, Ismail SA Involvement of the anti-inflammatory, anti-apoptotic, and anti-secretory activity of bee venom in its therapeutic effects on acetylsalicylic acid-induced gastric ulceration in rats. Toxicology 2019, 419: 11–23. 30885738 10.1016/j.tox.2019.03.003

[13] Gestwicki JE, Garza D Protein quality control in neurodegenerative disease. Prog Mol Biol Transl Sci 2012, 107: 327–353. 22482455 10.1016/B978-0-12-385883-2.00003-5

[14] He H, Li X, Yu H, Zhu S, He Y, Komatsu K, Guo D, et al. Gastroprotective effect of araloside a on ethanol-and aspirin-induced gastric ulcer in mice: involvement of H+/K+-ATPase and mitochondrial-mediated signaling pathway. J Nat Med 2019, 73: 339–352. 30523551 10.1007/s11418-018-1256-0

[15] Abdelfattah MS, Elmallah MI, Ebrahim HY, Almeer RS, Eltanany RM, Moneim AEA Prodigiosins from a marine sponge-associated actinomycete attenuate HCl/ethanol-induced gastric lesion via antioxidant and anti-inflammatory mechanisms. PloS One 2019, 14: e0216737. 31194753 10.1371/journal.pone.0216737 PMC6563954

[16] Mei X-T, Xu D-H, Xu S-K, Zheng Y-P, Xu S-B Zinc (II)–curcumin accelerates the healing of acetic acid-induced chronic gastric ulcers in rats by decreasing oxidative stress and downregulation of matrix metalloproteinase-9. Food Chem Toxicol 2013, 60: 448–454. 23933360 10.1016/j.fct.2013.07.075

[17] Munawar KS, Haroon SM, Hussain SA, Raza H Schiff bases: multipurpose pharmacophores with extensive biological applications. J Basic Appl Sci 2018, 14: 217–229.

[18] Da Silva CM, da Silva DL, Modolo LV, Alves RB, de Resende MA, Martins CV, de Fátima Â Schiff bases: a short review of their antimicrobial activities. J Adv Res 2011, 2: 1–8.

[19] Salga MS, Ali HM, Abdulla MA, Abdelwahab SI, ElhassanTaha MM, Synthesis YU Gastroprotective activities of some zinc (II) complexes derived from (E)-2-(1-(2-(piperazin-1-yl) ethylimino) ethyl) phenol and (E)-4-(1-(2-(piperazin-1-yl) ethylimino) ethyl) benzene-1, 3-diol Schiff bases against aspirin induced ulceration. Arab J Chem 2017, 10: S1578–S1589.

[20] Batra C, Synthesis CHKA Characterization and biological evaluation of quinazolinone substiuted benzothiazole via Schiff Base for antioxidant activity. Glob J Pharma Educ Res 2018, 3: 1–2.

[21] Murtaza S, Akhtar MS, Kanwal F, Abbas A, Ashiq S, Synthesis SS Biological evaluation of schiff bases of 4-aminophenazone as an anti-inflammatory, analgesic and antipyretic agent. J Saudi Chem Soc 2017, 21: S359–S372.

[22] Faghih Z, Neshat A, Wojtczak A, Faghih Z, Mohammadi Z, Varestan S Palladium (II) complexes based on Schiff base ligands derived from ortho-vanillin; synthesis, characterization and cytotoxic studies. Inorg Chim Acta 2018, 471: 404–412.

[23] Asif M Chemical characteristics, synthetic methods, and biological potential of quinazoline and quinazolinone derivatives. Int J Med Chem 2014, 2014: 395637. 25692041 10.1155/2014/395637 PMC4321853

[24] Jaisankar P, Swarnakar S, Chatterjee S, Verma S, Mandal M, Chaudhuri SR 3-indolyl furanoids as inhibitors of matrix metalloproteinase-9 for prevention of gastric ulcer and other inflammatory diseases. Google Patents 2018, 15: 1–7.

[25] Herowati R, Kartasasmita RE, Adnyana IK, Kartawinata TG Anti-inflammatory activities and gastric ulcer-inducing properties of tetraacetylquercetin and tetrapivaloylquercetin. J Math Fundam Sci 2016, 48: 252–262.

[26] Saremi K, Rad SK, Tayeby F, Abdulla MA, Karimian H, Majid NA Gastroprotective activity of a novel Schiff base derived dibromo substituted compound against ethanol-induced acute gastric lesions in rats. BMC Pharmacol Toxicol 2019, 20: 13. 30770761 10.1186/s40360-019-0292-z PMC6377749

[27] Yaul A, Pethe G, Deshmukh R, Aswar A Vanadium complexes with quadridentate Schiff bases. J Ther Anal Calorim 2013, 113: 745–752.

[28] Ghasemzadeh A, Jaafar HZ, Rahmat A Antioxidant activities, total phenolics and flavonoids content in two varieties of Malaysia young ginger (*Zingiber officinale* roscoe). Molecules 2010, 15: 4324–4333. 20657444 10.3390/molecules15064324 PMC6264263

[29] AlRashdi AS, Salama SM, Alkiyumi SS, Abdulla MA, Hadi AHA, Abdelwahab SI, Taha MM, et al. Mechanisms of gastroprotective effects of ethanolic leaf extract of Jasminum sambac against HCl/ethanol-induced gastric mucosal injury in rats. Evid Based Complement Alternat Med 2012, 2012: 786426. 22550543 10.1155/2012/786426 PMC3329065

[30] Mahmood A, Fouad A-B, Noor S, Wasman S, Hussain SF Anti-ulcerogenic effects of Nagilla sativa in ethanol-induced gastric injuries in rats. J Med Plants Res 2011, 5: 5577–5583.

[31] Shakir R, Ariffin A, Abdulla M Synthesis of new 2, 5-di-substituted 1, 3, 4-oxadiazoles bearing 2, 6-di-tert-butylphenol moieties and evaluation of their antioxidant activity. Molecules 2014, 19: 3436–3449. 24658568 10.3390/molecules19033436 PMC6271237

[32] Abdulla MA, Ali HM, Ahmed KA-A, Noor SM, Ismail S Evaluation of the anti-ulcer activities of *Morus alba* extracts in experimen-tally-induced gastric ulcer in rats. Biomedical Research-India 2009, 20: 35–39.

[33] Golbabapour S, Gwaram NS, Hassandarvish P, Hajrezaie M, Kamalidehghan B, Abdulla MA, Ali HM, et al. Gastroprotection studies of Schiff base zinc (II) derivative complex against acute superficial hemorrhagic mucosal lesions in rats. PloS One 2013, 8: e75036. 24058648 10.1371/journal.pone.0075036 PMC3772879

[34] Ketuly KA, Hadi AHA, Golbabapour S, Hajrezaie M, Hassandarvish P, Ali HM, Majid NA, et al. Acute toxicity and gastroprotection studies with a newly synthesized steroid. PloS one 2013, 8: e59296. 23516624 10.1371/journal.pone.0059296 PMC3596355

[35] Wong J-Y, Abdulla MA, Raman J, Phan C-W, Kuppusamy UR, Golbabapour S, Sabaratnam V Gastroprotective effects of Lion’s mane mushroom *Hericium erinaceus* (bull.:Fr.) Pers. (Aphyllophoromycetideae) extract against ethanol-induced ulcer in rats. Evid Based Complement Alternat Med 2013, 2013: 492976. 24302966 10.1155/2013/492976 PMC3835629

[36] Ketuly KA, Abdulla MA, Hadi HA, Mariod AA, Abdel-Wahab SI Anti-ulcer activity of the 9alpha-bromo analogue of Beclomethasone dipropionate against ethanol-induced gastric mucosal injury in rats. J Med Plants Res 2011, 5: 514–520.

[37] Corne S A method for the quantitative estimation of gastric barrier mucus. J Physiol (London) 1974, 242: 116–117. 4142046

[38] Robert A, Böttcher W, Golanska E, Kauffman GL Lack of correlation between mucus gel thickness and gastric cytoprotection in rats. Gastroenterology 1984, 86: 670–674. 6421648

[39] Sidahmed HMA, Azizan AHS, Mohan S, Abdulla MA, Abdelwahab SI, Taha MME, Hadi AHA, et al. Gastroprotective effect of desmosdumotin C isolated from *Mitrella kentii* against ethanol-induced gastric mucosal hemorrhage in rats: possible involvement of glutathione, heat-shock protein-70, sulfhydryl compounds, nitric oxide, and anti-helicobacter pylori activity. BMC Complement Altern Med 2013, 13: 183. 23866830 10.1186/1472-6882-13-183 PMC3765280

[40] Gornall AG, Bardawill CJ, David MM Determination of serum proteins by means of the biuret reaction. J Biol Chem 1949, 177: 751–766. 18110453

[41] Abdulla M, Al-Bayaty F, Younis L, Hassan MA Anti-ulcer activity of *Centella asiatica* leaf extract against ethanol-induced gastric mucosal injury in rats. J Med Plants Res 2010, 4: 1253–1259.

[42] Nordin N, Salama SM, Golbabapour S, Hajrezaie M, Hassandarvish P, Kamalidehghan B, Majid NA, et al. Anti-ulcerogenic effect of methanolic extracts from *Enicosanthellum pulchrum* (king) Heusden against ethanol-induced acute gastric lesion in animal models. PloS One 2014, 9: e111925. 25379712 10.1371/journal.pone.0111925 PMC4224391

[43] Malfertheiner P, Chan FK, McColl KE Peptic ulcer disease. The Lancet 2009, 374: 1449–1461. 10.1016/S0140-6736(09)60938-7 19683340

[44] Gwaram NS, Musalam L, Ali HM, Abdulla MA Synthesis of 2′-(5-Chloro-2-Hydroxybenzylidene) benzenesulfanohydrazide Schiff base and its anti-ulcer activity in ethanol-induced gastric mucosal lesions in rats. Trop J Pharma Res 2012, 11: 251–257.

[45] Ismail IF, Golbabapour S, Hassandarvish P, Hajrezaie M, Abdul Majid N, Kadir FA, Al-Bayaty F, et al. Gastroprotective activity of *Polygonum chinense* aqueous leaf extract on ethanol-induced hemorrhagic mucosal lesions in rats. Evid Based Complement Alternat Med 2012, 2012: 404012. 23365597 10.1155/2012/404012 PMC3544547

[46] Batran R, Al-Bayaty F, Abdulla M, Al-Obaidi M, Hajrezaei M, Hassandarvish P, Fouad M, et al. Gastroprotective effects of *Corchorus olitorius* leaf extract against ethanol-induced gastric mucosal hemorrhagic lesions in rats. J Gastroenterol Hepatol 2013, 28: 1321–1329. 23611708 10.1111/jgh.12229 PMC3842111

[47] Ibrahim M, Ali H, Abdullah M, Hassandarvish P Acute toxicity and gastroprotective effect of the Schiff base ligand 1H-indole-3-ethylene-5-nitrosalicylaldimine and its nickel (II) complex on ethanol induced gastric lesions in rats. Molecules 2012, 17: 12449–12459. 23090023 10.3390/molecules171012449 PMC6268460

[48] Hashim H, Mughrabi FF, Ameen M, Khaledi H, Ali HM Cytoprotective effect of benzyl N'-(5-Chloro-indol-3-yl-methylidene)-hydrazinecarbodithioate against ethanol-induced gastric mucosal injury in rats. Molecules 2012, 17: 9306–9320. 22864239 10.3390/molecules17089306 PMC6268369

[49] Mustafa IM, Hapipah MA, Abdulla MA, Ward TR Synthesis, structural characterization, and anti-ulcerogenic activity of schiff base ligands derived from tryptamine and 5-chloro, 5-nitro, 3, 5-ditertiarybutyl salicylaldehyde and their nickel (II), copper (II), and zinc (II) complexes. Polyhedron 2009, 28: 3993–3998.

[50] Ibrahim MY, Hashim NM, Dhiyaaldeen SM, Al-Obaidi MMJ, El-Ferjani RM, Adam H, Alkotaini B, et al. Acute toxicity and gastroprotection studies of a new schiff base derived manganese (II) complex against Hcl/ethanol-induced gastric ulcerations in rats. Sci Rep 2016, 6: 26819. 27229938 10.1038/srep26819 PMC4882520

[51] Salga MS, Ali HM, Abdullah MA, Abdelwahab SI, Hussain PD, Hadi AHA Mechanistic studies of the anti-ulcerogenic activity and acute toxicity evaluation of dichlorido-copper (II)-4-(2-5-Bromo-benzylideneamino) (ethyl) piperazin-1-ium phenolate complex against ethanol-induced gastric injury in rats. Molecules 2011, 16: 8654–8669.

[52] Golbabapour S, Gwaram NS, Al-Obaidi MMJ, Soleimani A, Ali HM, Abdul Majid N Schiff base metal derivatives enhance the expression of HSP70 and suppress BAX proteins in prevention of acute gastric lesion. Biomed Res Int 2013, 2013: 703626. 24298554 10.1155/2013/703626 PMC3835702

[53] Halabi MF, Shakir RM, Bardi DA, Al-Wajeeh NS, Ablat A, Hassandarvish P, Hajrezaie M, et al. Gastroprotective activity of ethyl-4-[(3, 5-di-tert-butyl-2-hydroxybenzylidene) amino] benzoate against ethanol-induced gastric mucosal ulcer in rats. PloS One 2014, 9: e95908. 24800807 10.1371/journal.pone.0095908 PMC4011731

[54] Brzozowski T, Konturek P, Konturek S, Brzozowska I, Pawlik T Role of prostaglandins in gastroprotection and gastric adaptation. J Physiol Pharmacol 2005, 56: 33–55. 16247188

[55] Takeuchi K Gastric cytoprotection by prostaglandin E2 and prostacyclin: relationship to EP1 and IP receptors. J Physiol Pharmacol 2014, 65: 3–14. 24622825

[56] Hajrezaie M, Golbabapour S, Hassandarvish P, Gwaram NS, Hadi AHA, Ali HM, Majid N, et al. Acute toxicity and gastroprotection studies of a new schiff base derived copper (II) complex against ethanol-induced acute gastric lesions in rats. PloS One 2012, 7: e51537. 23251568 10.1371/journal.pone.0051537 PMC3519725

[57] Ibrahim IAA, Abdulla MA, Hajrezaie M, Bader A, Shahzad N, Al-Ghamdi SS, Gushash AS, et al. The gastroprotective effects of hydroalcoholic extract of *Monolluma quadrangula* against ethanol-induced gastric mucosal injuries in Sprague Dawley rats. Drug Des Devel Ther 2016, 10: 93. 10.2147/DDDT.S91247 PMC4699547 26766904

[58] Singh A, Puri D, Kumar B, Singh SK Heat shock proteins: knowledge so far and its future prospects. Asian J Pharm Clin Res 2016, 9: 17–24.

[59] Kim HP, Morse D, Choi AM Heat-shock proteins: new keys to the development of cytoprotective therapies. Expert Opin Ther Targets 2006, 10: 759–769. 16981832 10.1517/14728222.10.5.759

[60] Reed JC Double identity for proteins of the Bcl-2 family. Nature 1997, 387: 773. 9194558 10.1038/42867

[61] Salama SM, Abdulla MA, AlRashdi AS, Ismail S, Alkiyumi SS, Golbabapour S Hepatoprotective effect of ethanolic extract of *Curcuma longa* on thioacetamide induced liver cirrhosis in rats. BMC Complement Altern Med 2013, 13: 56. 23496995 10.1186/1472-6882-13-56 PMC3605171

[62] Kerr JF, Wyllie AH, Currie AR Apoptosis: a basic biological phenomenon with wideranging implications in tissue kinetics. Br J Cancer 1972, 26: 239. 4561027 10.1038/bjc.1972.33 PMC2008650

[63] Sidahmed HMA, Vadivelu J, Loke MF, Arbab IA, Abdul B, Sukari MA, Abdelwahab SI Anti-ulcerogenic activity of dentatin from clausena excavata Burm. f. against ethanol-induced gastric ulcer in rats: possible role of mucus and anti-oxidant effect. Phytomedicine 2019, 55: 31–39. 30668441 10.1016/j.phymed.2018.06.036

